# Interannual Change Detection of Mediterranean Seagrasses Using RapidEye Image Time Series

**DOI:** 10.3389/fpls.2018.00096

**Published:** 2018-02-06

**Authors:** Dimosthenis Traganos, Peter Reinartz

**Affiliations:** ^1^Department of Photogrammetry and Image Analysis, German Aerospace Center Deutsches Zentrum für Luft—und Raumfahrt, Remote Sensing Technology Institute, Berlin, Germany; ^2^Department of Photogrammetry and Image Analysis, German Aerospace Center Deutsches Zentrum für Luft—und Raumfahrt, Remote Sensing Technology Institute, Wessling, Germany

**Keywords:** *Posidonia oceanica*, *Cymodocea nodosa*, Mediterranean seagrasses, Thermaikos Gulf, RapidEye, time series, change detection, Random Forests

## Abstract

Recent research studies have highlighted the decrease in the coverage of Mediterranean seagrasses due to mainly anthropogenic activities. The lack of data on the distribution of these significant aquatic plants complicates the quantification of their decreasing tendency. While Mediterranean seagrasses are declining, satellite remote sensing technology is growing at an unprecedented pace, resulting in a wealth of spaceborne image time series. Here, we exploit recent advances in high spatial resolution sensors and machine learning to study Mediterranean seagrasses. We process a multispectral RapidEye time series between 2011 and 2016 to detect interannual seagrass dynamics in 888 submerged hectares of the Thermaikos Gulf, NW Aegean Sea, Greece (eastern Mediterranean Sea). We assess the extent change of two Mediterranean seagrass species, the dominant *Posidonia oceanica* and *Cymodocea nodosa*, following atmospheric and analytical water column correction, as well as machine learning classification, using Random Forests, of the RapidEye time series. Prior corrections are necessary to untangle the initially weak signal of the submerged seagrass habitats from satellite imagery. The central results of this study show that *P. oceanica* seagrass area has declined by 4.1%, with a trend of −11.2 ha/yr, while *C. nodosa* seagrass area has increased by 17.7% with a trend of +18 ha/yr throughout the 5-year study period. Trends of change in spatial distribution of seagrasses in the Thermaikos Gulf site are in line with reported trends in the Mediterranean. Our presented methodology could be a time- and cost-effective method toward the quantitative ecological assessment of seagrass dynamics elsewhere in the future. From small meadows to whole coastlines, knowledge of aquatic plant dynamics could resolve decline or growth trends and accurately highlight key units for future restoration, management, and conservation.

## Introduction

Seagrasses are one of the most vital constituents of the Mediterranean coastal environment. Spanning a depth range between 0 and 45 m where there is enough light availability for their growth, these marine flowering plants play a major role in the great Mediterranean biodiversity of 18% of all known marine species (Coll et al., 2010). In addition to enhancing biodiversity, Mediterranean seagrasses contribute to a plethora of valuable ecosystem services (Costanza et al., 1997; Vassallo et al., 2013; Campagne et al., 2015) including nursery grounds (Giannoulaki et al., 2013), coastal erosion buffering (Pergent et al., 2012), bio-indicator (Mtwana Nordlund et al., 2016), and carbon sequestration (Fourqurean et al., 2012). Regarding the latter ecosystem service, Mediterranean seagrass meadows store the largest pools of the remineralization of organic carbon; nearly three times greater mean living biomass is present in the Mediterranean seagrasses than the global mean of nine seagrass bioregions (Fourqurean et al., 2012).

Despite their ecological value and being a conservation priority of national and international legislations, Mediterranean seagrasses have faced regression between 10 and 38% during the last 50 years (Tomas et al., 2005; Marbà et al., 2014; Telesca et al., 2015). This regression has been mainly attributed to anthropogenic activities including trawling, coastal artificialization, anchoring of heavy ships, dredging, and climate change (Leriche et al., 2006; Waycott et al., 2009; Jordà et al., 2012; Bonacorsi et al., 2013). The slow growth of 3–4 cm/yr of *P. oceanica* seagrass along with the sparsity of data on the distribution of Mediterranean seagrass habitats, mainly in the southern and eastern Mediterranean (Telesca et al., 2015), hamper any effort for their effective conservation management. The question then arises: how can we conserve something which grows slow, declines fast, and we have limited information on its spatial distribution?

The answer to the above question may lie in Earth observation. In contrast and somewhat parallel to the decreasing trend in coverage of Mediterranean seagrass meadows, satellite remote sensing technology has grown at an unprecedented pace, mainly since the end of the 1990s (Dekker et al., 2006). Advances in Earth observation have resulted from single sensors (e.g., Landsat 7 and 8, SPOT 4–7, QuickBird 2, WorldView 1–4) to constellations of satellites (e.g., Sentinel-2, Planet's RapidEye and Doves). This swarm of satellites images the Earth's surface with medium to high spatial (0.31–30 m), temporal (1–16 days), spectral, and radiometric resolution.

Applied to the coastal environment, spaceborne image archives allow multi-temporal analysis and change detection of submerged ecosystems which could in turn permit identification of possible degradation rates and boost conservation efforts of these problematic areas (Purkis and Roelfsema, 2015). Researchers have previously employed spaceborne time series to map seasonal to decadal change detection of seagrasses (Dekker et al., 2005; Knudby et al., 2010; Lyons et al., 2012; Pu et al., 2014; Roelfsema et al., 2014; Hossain et al., 2015). The time-series analysis of seagrass communities is as accurate as the classification algorithms in use (Palandro et al., 2003). More recently, machine learning algorithms (e.g., Random Forests, Support Vector Machines, k-nearest neighbors) have overruled simpler classification algorithms in the remote sensing literature (Gislason et al., 2006; Mountrakis et al., 2011). However, machine learning has been sparsely implemented in the quantitative assessment of coastal environments (Zhang, 2015).

In this study, our main aim is to combine recent advances in high spatial resolution sensors and machine learning algorithms to study the interannual dynamics of Mediterranean seagrasses by processing and analyzing a multispectral RapidEye time series in the Thermaikos Gulf, NW Aegean Sea (Greece) between 2011 and 2016. Featuring a great water transparency, small depth slope and thriving in two seagrass species, *Posidonia oceanica* and *Cymodocea nodosa* (Traganos and Reinartz, 2017; Traganos et al., 2017), the southeastern shelf Thermaikos Gulf comprises a suitable natural laboratory to apply our spaceborne change detection analysis. The spatio-temporal dynamics and sources of observed variations of Mediterranean seagrass habitats, namely the intertidal *Zostera noltii* and *C. nodosa* species, have been assessed before through spaceborne and airborne time series (Barillé et al., 2010; Garrido et al., 2013). The ecological status of the dominant and endemic in the Mediterranean, *P. oceanica*, however, has not been assessed yet via change detection analysis. RapidEye constellation of five satellites was the first to provide high spatial resolution data with a daily revisit time over the same area^1^. There have been few applications of RapidEye data in aquatic habitat mapping (Roessler et al., 2012; Giardino et al., 2015; Fritz et al., 2017). In general, temporally separated satellite image sequences over coastal areas acquired under different conditions (e.g., atmospheric, water column composition) can impede the change detection mapping of submerged habitats like seagrasses (Purkis and Roelfsema, 2015).

## Materials and methods

### Study site

**Figure 1:**
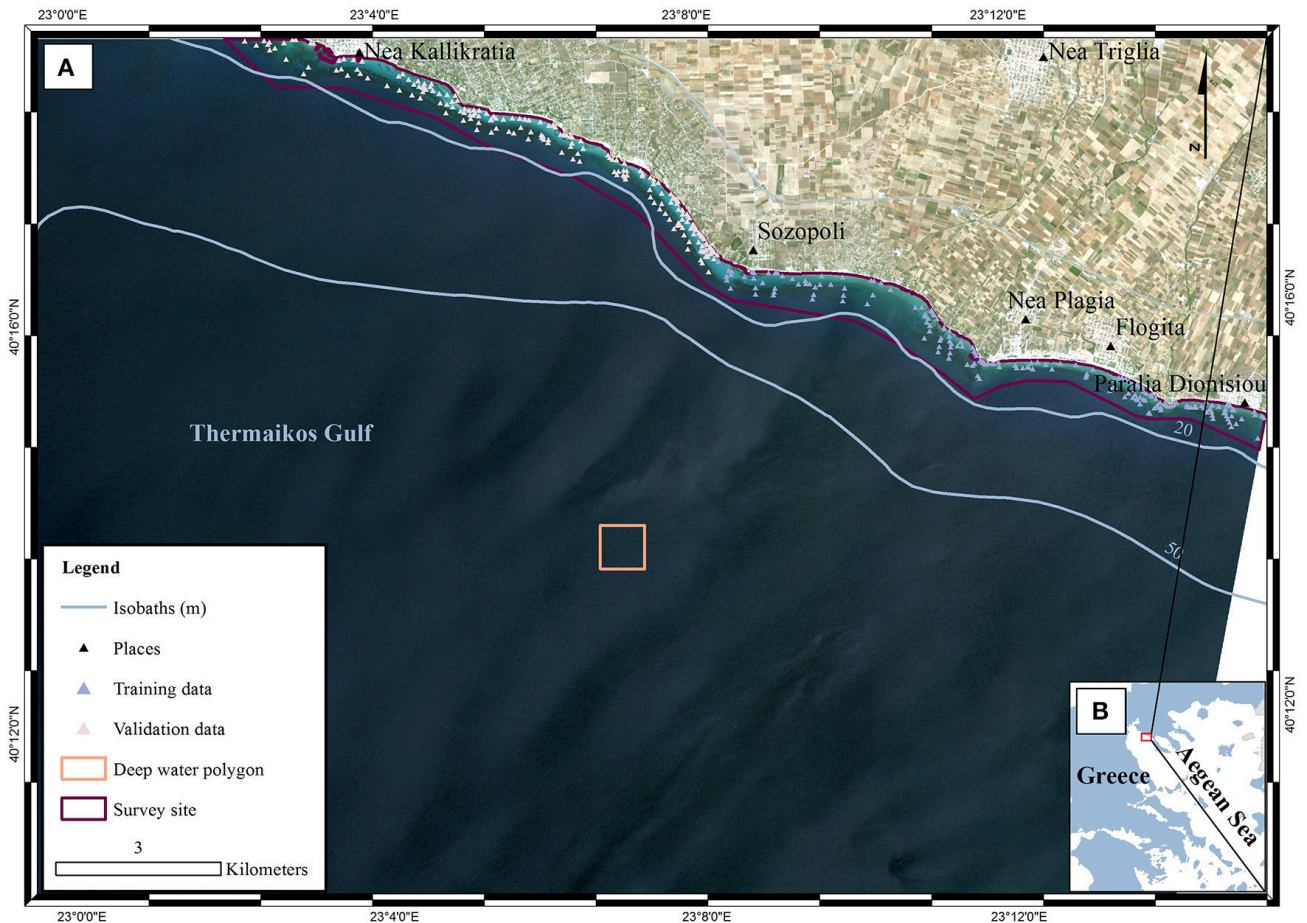
Location of survey site within **(A)** Thermaikos Gulf, **(B)** Aegean Sea, Greece. The displayed RapidEye imagery is a non-atmospherically corrected, true color (band 1 as blue, band 2 as green, band 3 as red) composite in UTM (zone 34) system/WGS84 projection. The imagery was acquired on 22/06/2016 (RE16 in text). The red polygon in **(B)** depicts the location of **(A)** within the Thermaikos Gulf. The deep water polygon represents a ~160 × 160 pixel window implemented in the water column correction of the image time series as it represents an area with very little water leaving radiance values in all three bands.

The study site is a submerged area of 888 hectares in the eastern Thermaikos Gulf, NW Aegean Sea, Greece (eastern Mediterranean Sea; Figure 1). The climate, oceanography, and hydrography of its water have been comprehensively described elsewhere (Poulos et al., 2000; Traganos and Reinartz, 2017). Satellite-derived mapping has revealed that the Thermaikos Gulf contains extensive beds of varying density of two seagrass species, *P. oceanica* and *C. nodosa*, between 1.4 and 16.5 m of depth (Traganos and Reinartz, 2017; Traganos et al., 2017).

The coastal system of the eastern Thermaikos Gulf administratively belongs to the Municipality of Nea Propontida. With a population of 36,500^2^, Nea Propontida hosts numerous socioeconomic activities including fishing, aquaculture, tourism, agriculture, industry, and trade directly or indirectly influence the marine environment. Furthermore, the administrative region of Central Macedonia, which contains Nea Propontida, features a total fishing catch of 11,869 t (18.5% of the total Greek fishing catch)^3^. Last but not least, 19.3% of the total citizens of the municipality are employed in the primary sector, while 66 factories and 91 hotels are running in the coastal region^2^.

### Satellite data

Planet's RapidEye constellation consists of five multispectral (five bands between 440 and 850 nm) satellites which collect high-spatial (5-m orthorectified pixel size) and temporal (daily off-nadir and 5.5 days at nadir) imagery. Although designed to operate for a minimum of seven years, RapidEye satellites have already collected an 8-year image archive. Fourty-seven Level 3A image tiles fell within the extent of our study site. These tiles are individual 25 × 25 km orthorectified imagery products with applied geometric, radiometric, and sensor corrections^1^. Based on a preliminary visual examination, we selected four from these image tiles (Table 1) which satisfied optimum conditions for remote sensing of optically shallow environment (e.g., cloud-, sunglint- and skyglint-free, no or low concentration of water column constituents, same season). The four images comprise a time series which spans the years between 2011 and 2016. For ease, we will refer to the four images with the abbreviation RE (stands for RapidEye) and the two last digits from the year of acquisition; RE11, RE12, RE15, RE16.

**Table 1:** Characteristics of the high spatial resolution satellite imagery and respective input parameters for running FLAASH module.

**Satellite imagery**	**Scene acquisition date (dd/mm/year)**	**Scene acquisition time (local)**	**Atmospheric model**	**Aerosol model**
RE11	13/05/2011	13:23	SAS^1^	Maritime
RE12	24/09/2012	13:16	MLS^2^	Rural
RE15	18/09/2015	12:57	MLS	Maritime
RE16	22/06/2016	12:53	MLS	Maritime

*RE, RapidEye, 5 × 5 m pixel, blue, green, red, red edge, nir*.

1 *Sub-Arctic Summer*.

2 *Mid-Latitude Summer*.

### Field data

The field data collection is described in Traganos and Reinartz (2017). We collected these field data, namely habitat-related points with associated coordinates and bathymetry data, during a boat-based survey between 10 and 13 July 2016. Furthermore, we added more data points following interpretation of the high resolution RapidEye imagery. We selected data points that have indicated the same habitat within the 5-year span of our time series analysis. Four-hundred data points (Figure 1) were used for both training and validation of the machine learning classifier implemented here. In the bathymetry estimation step, we employed the image chronologically closest to the field data collection, RE16, to develop a pixel-based bathymetry map for subsequent use in the water column correction step.

### Methodology

**Figure 2:**
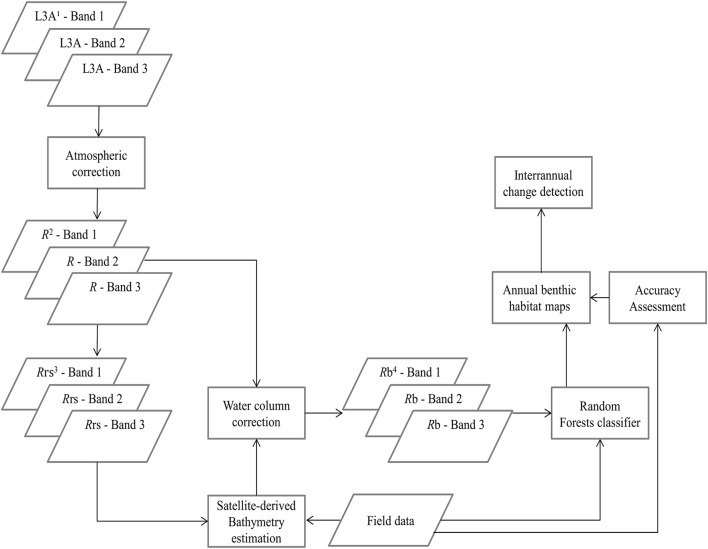
Schematic representation of the methodology. ^1^L3A ortho products are the initial radiometric, sensor, and geometrically corrected RapidEye images in UTM/WGS1984 projection, ^2^*R* represent atmospherically-corrected (FLAASH module), at-water surface reflectances, ^3^*R*_rs_ are remote sensing reflectances, transformed from *R* using Equation (1), ^4^*R*_*b*_ are water-column-corrected, bottom reflectances using the analytical model of Maritorena et al. (1994).

**Figure 3:**
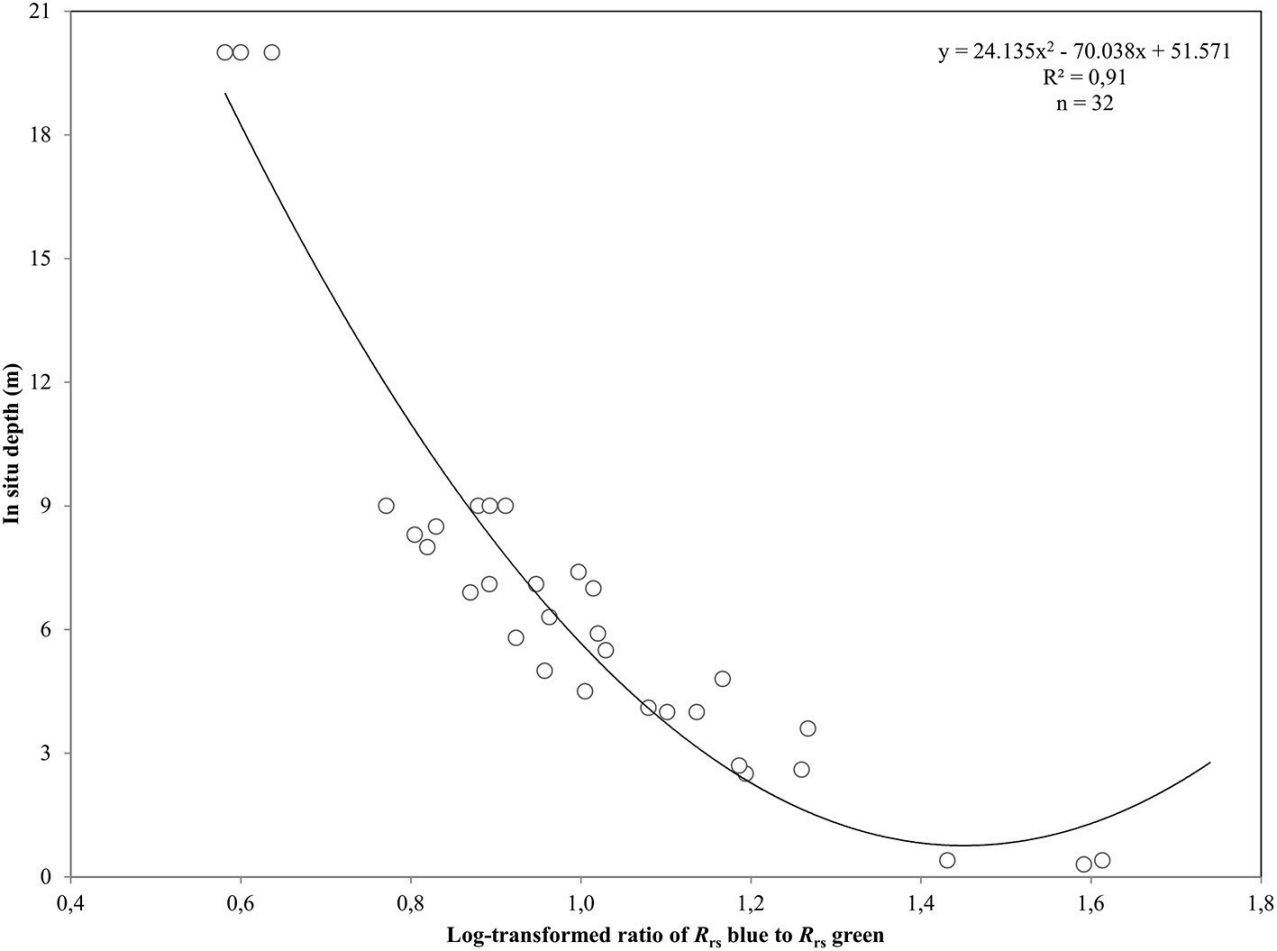
Polynomial regression between the log-transformed ratio of blue and green remote sensing reflectances, *R*_rs_, and *in situ* depth measurements from the Thermaikos survey site. The shown polynomial equation was implemented to estimate the bathymetry map displayed in **(C)** of Figure 4.

**Figure 4:**
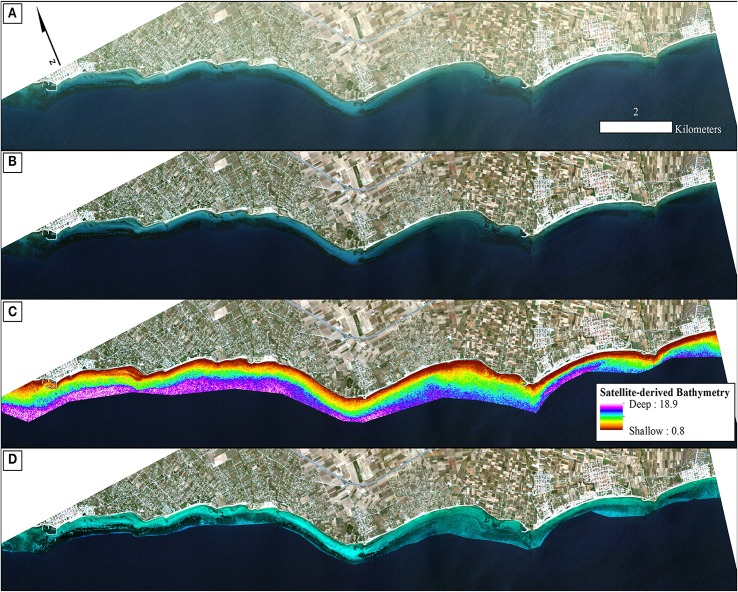
Methodological steps from atmospheric to water column correction in order of successive processing. All four panels are true color RapidEye image composites (22/06/2016; RE16 in text) projected in UTM (zone 34) system/WGS84. **(A)** Non-atmospherically corrected composite. **(B)** Atmospherically-corrected composite using the FLAASH module. **(C)** Satellite-derived Bathymetry map of the survey site draped over the atmospherically-corrected composite of **(B)** using the site-specific polynomial algorithm of Equation (2) as shown on Figure 3. We applied a 5 × 5 low-pass filter on the initial ratio-derived bathymetry (not shown here) to reduce potential noise which would be transferred to the water-column corrected product. **(D)** Water-column corrected composite following application of the water column correction algorithm of Maritorena et al. (1994) draped over the atmospherically-corrected composite of **(B)** and masked using the optically deep limit of 16.5 m to enhance bottom features and potentially increase classification accuracies.

To derive quantitative information from coastal image time series using remote sensing, the analyst has to address the interference of the atmospheric, air-water interface, and water column by applying the same processing protocol on all satellite images which comprise the image time series. Figures 2, 3 show a schematic and visual representation of the processed protocol herein, respectively, until the classification step. The pre-classification steps which we followed in the present study included: (1) atmospheric correction to derive at-water surface reflectances without atmospheric interference (Figure 4B), (2) bathymetry estimation for use in the water column correction step (Figure 4C), and (3) water column correction to derive bottom reflectances without water column interference (Figure 4D). The classification step concerned the use of Random Forests (RF), an ensemble supervised classification algorithm which has received small attention in the remote sensing of optically shallow environments. The accuracy assessment of the RF-derived results was performed using the traditional error matrices reporting overall, producer, user, and kappa accuracies (Table 2). Finally, we conducted the interannual change detection of the two Mediterranean seagrass species, *P. oceanica* and *C. nodosa*, on the basis of area change and related trend throughout the 4 years.

**Table 2:** Error matrices of the four water-column corrected bottom reflectance images.

**Classes**	***Cymodocea nodosa***	***Posidonia oceanica***	**Rocky algae**	**Sand**	**Total**	**User accuracy (%)**
*Cymodocea nodosa*	16	1	2	2	21	76.2
*Posidonia oceanica*	3	46	3	1	53	86.8
Rocky algae	2	3	38	0	43	88.4
Sand	29	0	7	47	83	56.6
Total	50	50	50	50	200	
Producer accuracy	32	92	76	94		
**2011—OVERALL ACCURACY: 73.5%**						
*Cymodocea nodosa*	31	4	0	1	36	86.1
*Posidonia oceanica*	0	45	0	0	45	100
Rocky algae	0	0	37	0	37	100
Sand	19	1	13	49	82	59.8
Total	50	50	50	50	200	
Producer accuracy	62	90	74	98		
**2012—OVERALL ACCURACY: 81%**						
*Cymodocea nodosa*	30	11	0	0	41	73.2
*Posidonia oceanica*	1	38	1	0	40	95
Rocky algae	0	1	40	1	42	95.2
Sand	19	0	9	49	77	63.6
Total	50	50	50	50	200	
Producer accuracy	60	76	80	98		
**2015—OVERALL ACCURACY: 78.5%**						
*Cymodocea nodosa*	23	1	0	1	25	89.3
*Posidonia oceanica*	6	49	3	0	58	79.3
Rocky algae	0	0	43	0	43	100
Sand	21	0	4	49	74	55
Total	50	50	50	50	200	
Producer accuracy	46	98	86	98		
**2016—OVERALL Accuracy: 82%**						

*Date of each imagery is provided on the low left of each error matrix before Overall accuracy*.

#### Atmospheric correction

The first step of the pre-classification procedure was the atmospheric correction. We implemented the Fast Line-of-sight Atmospheric Analysis of Spectral Hypercubes (FLAASH) algorithm to correct the atmospheric interference on all RE images. The input parameters to run the FLAASH module are described in Table 1. All aerosol models were set as Maritime type except from the RE12 imagery for which experiments using the same type resulted to negative reflectances, therefore, we used the Rural type. The FLAASH module resulted to at-water surface reflectances, *R*, of all 5 RE bands (Figure 4B). The positional accuracy on the initial Level 3A RE image tiles was found to be adequate, hence we performed no additional coregistration on the four RE images which is a necessary step otherwise due to the pixel-based approach of the present study. In addition, due to our preliminary visual examination of the Level 3A products, no evident sunglint was found in the at-water surface reflectance composites.

#### Bathymetry estimation

Bathymetry knowledge of a coastal site is crucial to reduce reflectance changes due to water column attenuation and variable depth. Differences between reflectances of coexisting submerged habitats can hinder their detection through remote sensing. As a result of the shading which occur within the canopy, *P. oceanica* seagrass exhibit lower reflectances than its seaward neighbor, optically deep water (Dekker et al., 2006). Coupled with the high reflectances of a submerged sandy substrate, *P. oceanica* would look deeper than the sandy substrate at the same true depth (Traganos and Reinartz, 2017). Remote sensing researchers have developed band ratios to tackle the aforementioned issues and to measure bathymetry (Lyzenga, 1978; Dierssen et al., 2003; Stumpf et al., 2003). The basic assumption of band ratios is that the reflectance ratio in these bands remains constant irrespectively of the submerged environment.

To further eliminate interference at air-water interface, we chose to retrieve pixel-based bathymetry from the remote sensing reflectance, *R*_rs_, which is also less sensitive to water column properties (Mobley, 1994). We derived *R*_rs_ from at-water surface reflectances, *R*, using

(1) $$R_{rs}=\frac{tR}{Q}$$

where *t* is the transmittance of *E*_d_, spectral downwelling plane irradiance, and *L*_u_, spectral upwelling radiance, through the air-water interface and was calculated as 0.54 by Mobley (1994). *Q* factor is the ratio of *E*_u_, spectral upwelling plane irradiance, and *L*_u_ just beneath the water surface and depends on the type and depth of the bottom and the wavelength. We chose the π value for *Q* factor for the calculations of *R*_rs_, which is the theoretical value for Lambertian surfaces (Dierssen et al., 2003). We mapped the bathymetry of our survey site using the log-ratio of *R*_rs_ blue to *R*_rs_ green at 475 and 555 nm from RE16, respectively. We used RE16 because it is closer chronologically to our *in situ* data acquisition of July 2016. The first two RapidEye bands are attenuated less in the water column than the ones in the red, red edge and NIR. Thus, they comprise the ideal contestants to develop a second-order polynomial after plotting their ratio against *in situ* estimated bathymetry, *Z*

(2) $$Z=24.135x^{2}-70.038x+51.571$$

(3) $$x=\text{ln}\left(\frac{R_{\text{rs}}(475)}{R_{\text{rs}}(555)}\right)$$

which explained >91% of the variation (*p* < 0.001) in estimated bathymetry in 32 points (Figure 3) which spanned the whole depth range of habitat presence in our survey site. The site-specific algorithm of (2) was subsequently employed to create a pixel-based bathymetry map (Figure 4C). This bathymetry was further smoothed with a 5 × 5 low pass filter to reduce local variation and unwanted noise which would impede water column correction and possibly decrease classification accuracy. Based on the satellite-derived bathymetry and the findings of Traganos and Reinartz (2017), we applied an optically deep water mask utilizing the depth limit of 16.5 m to enhance submerged features in the classification step.

#### Water column correction

The water column correction step is vital to retrieve bottom reflectances from at-water surface reflectances. Here, we employ the approximate analytical solution of Maritorena et al. (1994) for optically shallow waters.

(4) $$R(\lambda )=R_{\infty }\left(\lambda \right)+\left(R_{\text{b}}\left(\lambda \right)-R_{\infty }\left(\lambda \right)\right)\exp\left(-2K_{\text{d}}\left(\lambda \right)Z\right)$$

where *R(*λ*)* is the atmospherically-corrected at-water surface reflectance composite of Atmospheric Correction (Figure 4B); *R*_∞_(λ) is the reflectance over an infinitely deep water column; *R*_b_(λ) is the bottom reflectance (Figure 4D); *K*_d_(λ) is the operational attenuation coefficient which expresses the attenuation of both upwelling and downwelling stream as these are originating from the seabed and from the water column (Maritorena et al., 1994). Ideally, *R*_∞_(λ) and *K*_d_(λ) are estimated using *in situ* optical measurements and radiative transfer simulations. In the absence of these, we used image-based techniques and existing measurements (Traganos and Reinartz, 2017). For the infinitely deep water column reflectance, *R*_∞_(λ), we extracted mean values from the deep water polygon of Figure 1A from the at-water surface reflectance RE16 composite, mean values which composed *R*_∞_(λ) for all four images of the RE time series. For the *K*_d_(λ), we used image-based calculated values for the water column corrections of all four dates from Traganos and Reinartz (2017) who used Bierwirth et al. (1993) approximations in the same area. Bottom reflectances (Figure 4D) were calculated for every pixel of the first three bands of the atmospherically corrected, at-water surface reflectance composites of all four RE images. We selected RE bands 1, 2, and 3 at 475, 555, and 658 nm since seagrasses and, generally, underwater habitats cannot be detected by wavelengths past 680 nm due to the great attenuation of pure water (Kirk, 1994). Last but not least, to save valuable space in the remaining text, we will refer to each quantity which is wavelength-dependent without its wavelength notation except when it is needed i.e., to discriminate between two quantities.

#### Random forest classification

The machine learning approach of Random Forests (RF) comprises an ensemble supervised classification algorithm that implements multiple self-learning decision trees to handle collinearity and, more significantly, non-linearity between predictor variables. Developed by Breiman (2001), RF are based on the assumption that different independent tree predictors give wrong predictions in different regions. By combining the results of the predictions, RF improve the efficiency of the model. Every decision tree in the implemented RF algorithm here is trained with a bootstrapped sample of the training data and at every split node, a subset of randomly selected features is utilized. The outputs are then combined by a simple majority vote. Generally, RF are robust against overtraining and noisy data in addition to providing good results with relatively small datasets (Gislason et al., 2006). Three parameters must be set before running the RF classifier: (a) the number of decision trees (*k*), (b) the number of randomly selected features (*n*_r_), and (c) the split selection. We selected 100 trees to run all RF experiments as they featured the best results out of a plethora of runs with different number of trees. In addition, we chose two for the number of *n*_r_ as well as the Gini Index for the measurement of the best split selection. We parameterized and ran all RF experiments using the EnMAP-Box software (van der Linden et al., 2015). All the experiments were performed using the bottom reflectance composites, *R*_b_ and 400 training and validation data for all four classes: (a) *C. nodosa* seagrass, (b) *P. oceanica* seagrass, (c) Rocky algae, and (d) Sand. The training and validation data were split equally into 50 data points per class for both training and subsequent validation through accuracy assessment.

#### Accuracy assessment

We used the error matrices (Table 2) to validate the results of the Random Forest classifications. As discussed in Random Forest Classification section, 50 data points per class were used to validate the RF classifier. The error matrix contains a square array of rows and columns where each of them represents one habitat class in the classification. Each cell in this matrix is the number of classified training samples, while the rows comprise classified training data and the columns are validation data for the assessment of the classified data. The error matrix outputs the overall, producer, and user accuracy (Congalton, 1991). The overall accuracy is the ratio of the number of correctly classified validation samples to the total number of validation data (200 in our study). On one hand, the producer accuracy expresses the number of correctly classified validation data in one class divided by the total number of validation data in the same class (50 in our study). On the other hand, the user accuracy corresponds to the number of correctly classified validation in one class divided by the total number of validation data that were classified in the same class. Although the producer accuracy is a solid statistical value for the creator of the habitat map (the remote sensing scientist as the case in point), the user accuracy is more vital from a management point of view as it reports the quantitative probability for the tangible presence of the habitat in the studied region i.e., *P. oceanica* and *C. nodosa* seagrass meadows.

#### Change detection

The ultimate aim of this study is to study the interannual change of two Mediterranean seagrasses, *P. oceanica*, and *C. nodosa*, exploiting the high spatial resolution of the RapidEye satellite constellation and the theoretical superiority of the machine learning classifier of Random Forests. Here, the interannual seagrass change detection (2011, 2012, 2015, and 2016) is conducted on an area change basis following the machine learning classification of water column corrected RapidEye composites. We report the area change throughout the 5-year time series in hectares in addition to the per-pixel gain, no change and loss between 2011 and 2016. A standard linear regression and associated slope coefficient are implemented to show approximate trends in area over time as well.

## Results

### Pre-classification steps

All pre-classification steps are displayed schematically in Figure 2 and visually in Figure 4. It is visually apparent that both the atmospheric and water column correction in Figures 4B,D, respectively, enhance bottom features following the increase of the seabed spectral variability from Figure 4A (initial top-of-the-atmosphere reflectance composite).

**Figure 5:**
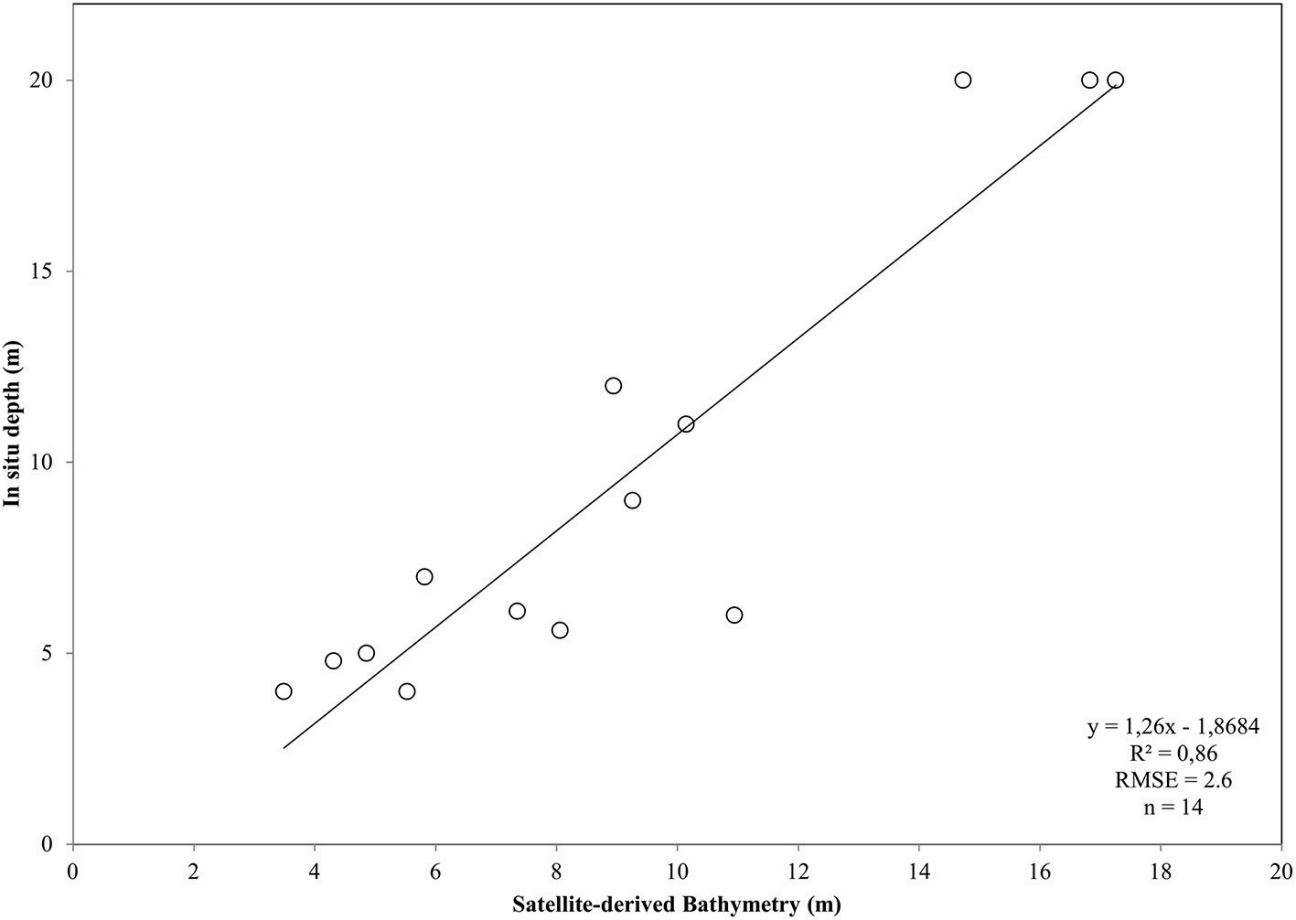
Plot of Satellite-derived Bathymetry (SDB) vs. *in situ* measured depth for the validation of the bathymetry map of the Thermaikos Gulf (Figure 4C). SDB was derived from Equation (2). Regressed SDB have been previously smoothed with a 5 × 5 low pass filter to reduce unwanted noise.

After converting the atmospherically-corrected at-water surface reflectances, *R* to *R*rs (1), we developed a site-specific polynomial algorithm (2) using the log-transformed ratio of the blue to green RapidEye bands of the RE16 image (3) to map bathymetry, *Z*, in our site (Figure 4C). The depth of the eastern coast of the Thermaikos Gulf spanned the depth range between 0.8 and 18.9 m with a mean depth of 7.7 m and a mean slope of 5.4°. The validation of the Satellite-derived Bathymetry was conducted using 14 *in situ* depth points and revealed an r-squared value of 0.86 with a root mean square error (RMSE) of 2.6 m (Figure 5). It is worth noting that from the 32 points used in the bathymetry estimation, 15 were measured above *P. oceanica* beds, 3 over *C. nodosa* beds, 11 over sandy seabed, and 3 over rocky seabed with photophilous algae. On the other hand, from the 14 points used in the bathymetry validation, 8 were over *P. oceanica* beds and 6 over sandy seabed.

Employing Equation (4), we performed water column correction for every pixel in all RE images. Equation (4) takes the at-water surface reflectance, *R*, the per-pixel Satellite-derived bathymetry, *Z*, the reflectance of an optically deep column, *R*_∞_, and, finally, the diffuse attenuation coefficient, *K*_d_, as inputs and outputs bottom reflectance, *R*_b_. The reflectances of an optically deep column were determined based on 25,599 pixels within the deep water polygon of Figure 1; *R*_∞_ (475) = 0.033, *R*_∞_ (555) = 0.024, and *R*_∞_ (658) = 0.017. As mentioned in section Water Column Correction, *K*_d_ values were selected for the whole time-series from Figure 11 in Traganos and Reinartz (2017); *K*_d_ (475) = 0.067, *K*_d_ (555) = 0.078, and *K*_d_ (658) = 0.134 (in m^−1^ as they are calculated based on the unitless *R* and depth).

### Random forest classification

**Figure 6:**
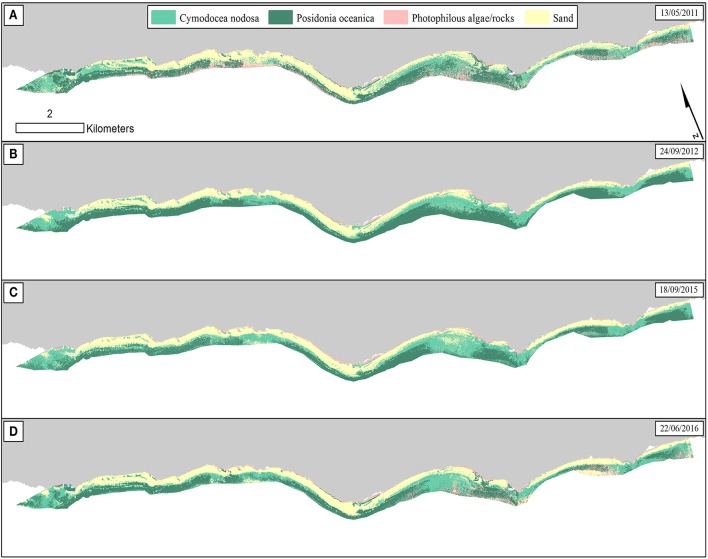
Classified water-column-corrected RapidEye composites from the 4 years using Random Forest machine learning classifier (100 trees). The frames on the upper right of each panel indicate the date of each RapidEye image. **(A)** RE11—Overall accuracy: 73.5%. **(B)** RE12—Overall accuracy: 81%. **(C)** RE15—Overall accuracy: 78.5%. **(D)** RE16—Overall accuracy: 82%.

We employed the Random Forest machine learning classifier on all bottom reflectance images which comprised the studied time series (Figure 4D). The results of the random forest classifications are presented in Figure 6. The accuracy assessment of the classification results for all images and habitats is presented in Table 2 with the form of four error matrices, one for each image from RE11 to RE16. All experiments were run using 100 trees. The two Mediterranean seagrasses under study here, *P. oceanica* and *C. nodosa*, showed a mean producer accuracy of 89 and 50%, respectively, with a mean user accuracy of 91.6 and 63.1%, correspondingly. From the whole time-series, *P. oceanica* seagrass was more accurately classified in RE16 (Figure 6D; 98% producer accuracy) and RE11 (Figure 6A; 92% producer accuracy), but less accurately identified in the same images according to the user accuracy of 84.5% of the former and 86.8% of the latter. The best user accuracies concerning *P. oceanica* were produced for the RE12 (Figure 6B; 100%) and the RE15 (Figure 6C; 95%).

As regards to *C. nodosa* species, RF correctly classified it to 62% and 60% producer accuracies in RE12 (Figure 6B) and RE15 (Figure 6C), while the former exhibited the second best user accuracy of 86.1% following the 92% of RE16 (Figure 6D). Generally, RE12 featured the second best overall accuracy (81%), marginally behind RE16 (82%), but possessed the best mean producer and user accuracies of the two seagrass habitats (76 and 93%, correspondingly). In the contrary, RE11 revealed the worst results with the worst overall accuracy of 73.5%, worst mean producer accuracy (62%), worst mean user accuracy (81.5%). Generally, the error matrices indicate that errors in both producer and user accuracies in all four images are mainly attributed to confusion between the two seagrasses and less with sandy or rocky seabed.

Based on the classified water column corrected RE16 composite, *P. oceanica* seagrass meadows covered an area of 264 ha in depths between 0.8 and 17.9 m, with an average depth presence of 8 m. On the other hand, *C. nodosa* beds covered 242 ha and were spread between depths of 0.8 and 16.1 m, with a mean depth presence of 5.8 m.

### Change detection

**Figure 7:**
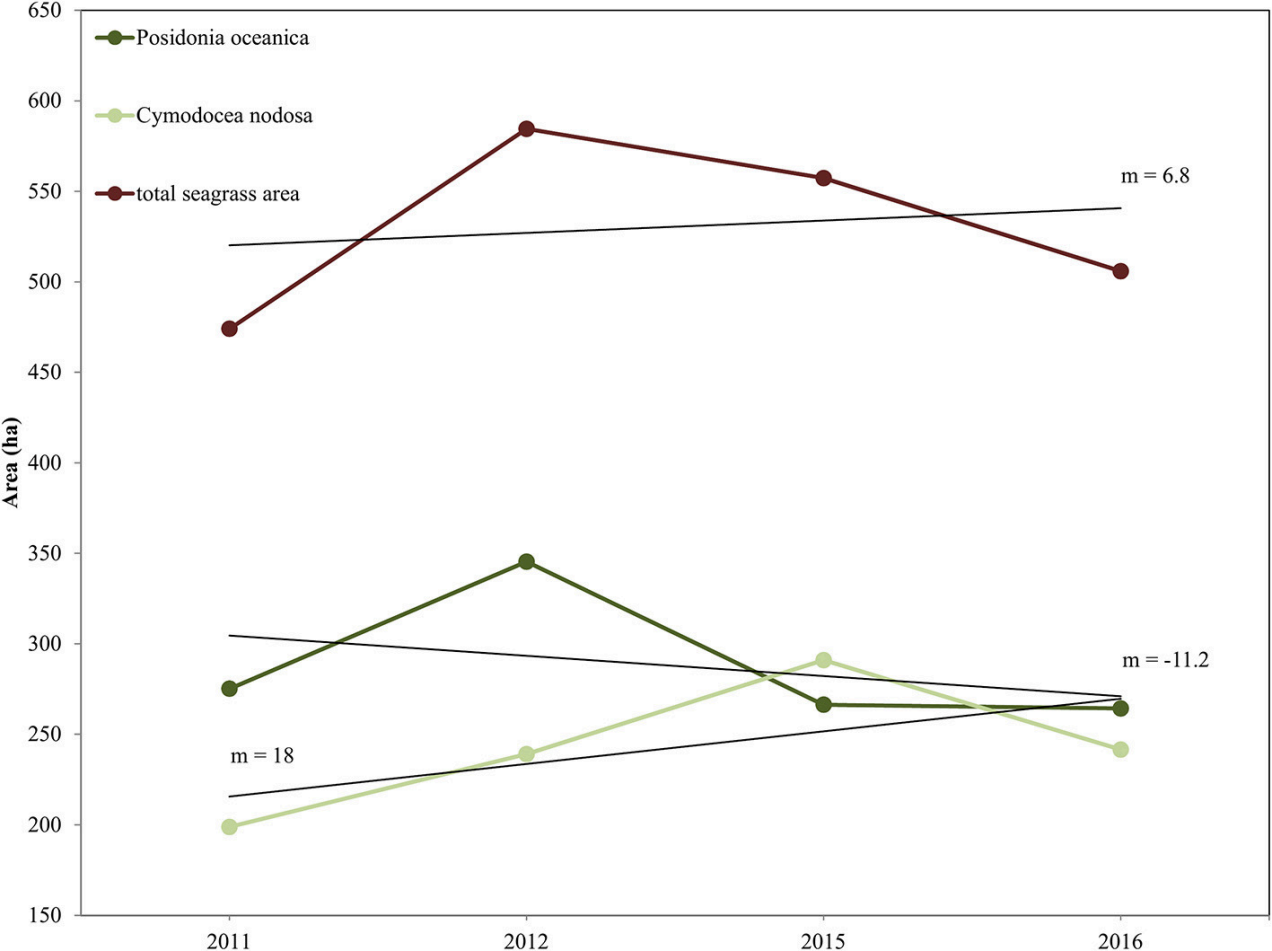
Interranual change detection of seagrasses in the Thermaikos Survey site between 2011 and 2016 using RapidEye satellite images. The trajectory plot displays change of area (in hectares; y-axis) over the years (x-axis) of *Posidonia oceanica* and *Cymodocea nodosa* species, and of total seagrass area. Linear regression black lines (m = slope) show approximate trend in area between 2011 and 2016. *Posidonia oceanica* seagrass is decreasing at 11.2 ha/yr, *Cymodocea nodosa* seagrass is increasing at 18 ha/yr, while total seagrass area is expanding at 6.8 ha/yr.

**Figure 8:**
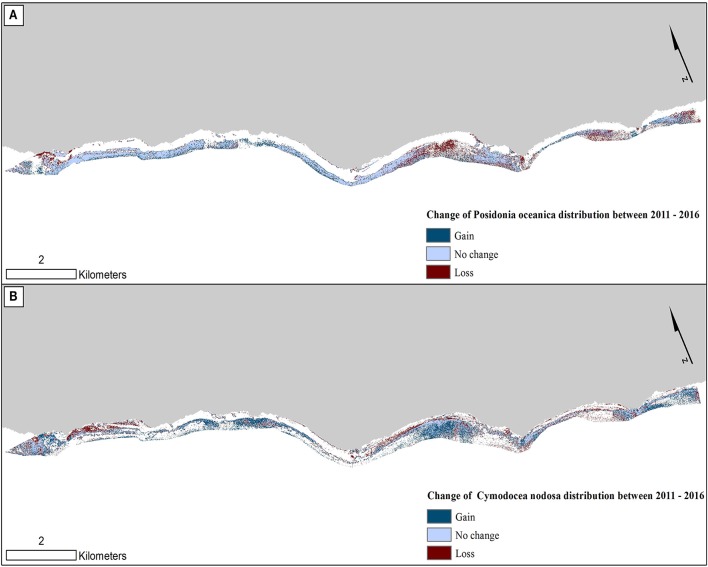
Change in seagrass distribution in the Thermaikos survey site between 2011 and 2016 for **(A)** *Posidonia oceanica* and **(B)** *Cymodocea nodosa*. Between 2011 and 2016, *P. oceanica* seagrass meadows have declined by 4.1%, while *C. nodosa* seagrasses have increased by 17.7%.

We report the interannual change detection of *P. oceanica* and *C. nodosa* seagrasses here as change of their extent (Figures 7, 8) following random forest classification of all four RE images (Figure 6). Figure 7 shows the areas of both seagrasses and total seagrass area in each of the four studied years in addition to indicating change trends at species and total level (black lines). We observe that *P. oceanica* area declined by 4.1% (from 275 to 264 ha) between 2011 and 2016, while its declining trend was 11.2 ha/yr. On the contrary, *C. nodosa* area increased by 17.7% (from 199 to 242 ha), while its increasing trend was 18 ha/yr. Overall, therefore, the area of seagrasses in the Thermaikos Gulf increased by 6.8% (474 to 506 ha) between 2011 and 2016, with an increasing trend of 6.8 ha/yr. The highest decrease of *P. oceanica* seagrass was displayed between 2012 and 2015 (−29.7%), while the highest decrease of *C. nodosa* seagrass was shown between 2015 and 2016 (−20.5%), the same period where we observe the greatest decrease of total seagrass area (−10.2%).

Figure 8 reveals the change of both studied Mediterranean seagrasses on a gain, no change and loss basis between 2011 and 2016. The greatest losses of *P. oceanica* meadows are depicted on the shallow northwesternmost part and the southeastern part of the center of our survey site (Figure 8A). On the other hand, while *C. nodosa* exhibit gains throughout the extent of the Thermaikos Gulf, its highest regression is also shown in the shallower seabed of the northwesternmost part. The importance of Figure 8 lies on its capacity to show possible losses of *P. oceanica* seagrass area attributed to gains of *C. nodosa* area. This is indeed the observation in the aforementioned area of the highest regression of *P. oceanica* species (center of our survey site; Figure 8) in addition to the southeasternmost extent of the Thermaikos Gulf site.

## Discussion

### Change detection

The main objective of this study was to evaluate whether the application of machine learning algorithms, namely Random Forests, on a time-series of high resolution satellite images, namely RapidEye, was effective for mapping the interranual change detection of two Mediterranean seagrasses, *P. oceanica* and *C. nodosa*, in 888 submerged hectares of the Thermaikos Gulf, NW Aegean Sea, Greece between 2011 and 2016 (total of four images). As attested by Figure 7, our main findings reveal that the distribution of *P. oceanica* seagrass meadows have declined by 4.1% with a decreasing trend of 11.2 ha/yr, while *C. nodosa* beds have increased by 17.7% with a gain trend of 18 ha/yr. Generally, total seagrass area increased by 6.3% at a +6.8 ha/yr trend throughout the 5 years. Approximate trends of seagrass distribution change are indicated by the slope coefficient of a standard linear regression between seagrass area and related years (Figure 7). This method of presenting remotely sensed time series of seagrass was utilized efficiently in 200 km^2^ of the Eastern Banks, Moreton Bay, Australia (Lyons et al., 2013).

To the best of our knowledge, the studied regression of 4.1% of *P. oceanica* meadows between 2011 and 2016 is the first report of regression of this particular seagrass species in the Greek seas and one of the first reports in the whole Eastern Mediterranean, a poorly mapped area. It is also in line with the reported trends in the Mediterranean and globally. Telesca et al. (2015) estimated an average regression of 10.1% for the whole extent of the Mediterranean basin during the past 50 years, which further increased to 33.6% for areas with existing historical information; Greece lacks this significant information. Marbà et al. (2014) further estimated that between 13 and 38% of initial *P. oceanica* meadows have been lost since 1960, with a decreasing trend of 1.74%/yr. On assessing 215 studies worldwide, Waycott et al. (2009) has shown that since 1990, seagrass grounds are disappearing at a median rate of 7%/yr, a 7-fold increase from the median rate of 0.9%/yr before 1940.

Based on the ecological value of *P. oceanica*, its reported regression of 4.1% in the Thermaikos Gulf translates into loss of the relevant ecosystem services which it provides in the broader region, including protection from coastal erosion, carbon sequestration, nursery grounds, and nutrient cycling among others. More specifically, the related economic loss to the declining rate of *P. oceanica* of 11.2 ha/yr is 19.264 million €/yr (Vassallo et al., 2013). This regression is more alarming due to the slow growth of *P. oceanica* meadows and the existing pressure from climate change. Mortality rates of *P. oceanica* seagrass are expected to increase 3-fold with an increase of 3°C in maximum annual seawater surface temperature (SST_max_) (Marbà and Duarte, 2010). In addition, the temperature of 28°C is the critical SST_max_, above which *P. oceanica* functional losses accelerate. Study of the specific drivers of the observed *P. oceanica* regression is out of our scope in the present study. Future studies, however, of the temporal dynamics of SST_max_ and the extent of *P. oceanica* seagrass meadows could unravel the underlying causes of their regression in the Thermaikos Gulf and elsewhere in the Mediterranean.

On the other hand, *C. nodosa* seagrass area faced a 17% increase of its extent, gaining 43 hectares between 2011 and 2016. We could attribute part of this increase to a combination of two physical factors. First, *C. nodosa* is a fast-growing seagrass with a reported rate of horizontal growth of up to 2 m/yr. Second, the decline in terms of *P. oceanica* seagrass allowed the fast recolonization of its regressed beds by the former seagrass, causing the expansion of its area. This substitution of *P. oceanica* by *C. nodosa* between 2011 and 2016 is particularly observed in the shallower parts of the northwesternmost, the middle and the southeasternmost regions of the Thermaikos Gulf (Figure 8).

Temporal dynamics of *C. nodosa* distribution have been sparsely studied elsewhere. *C. nodosa* populations have shown a progression of up to 42% between 1994 and 2011 in the Western Mediterranean, following a regression of 49% between 1973 and 1994 (Garrido et al., 2013). Furthermore, *C. nodosa* seagrass has been found to re-colonize the shallower regressed beds of *P. oceanica* seagrass (Montefalcone et al., 2007). All in all, *C. nodosa* seagrass competes with *P. oceanica* in terms of its expansion, while it is also considered as a significant step in the ecological succession prior to beds of the latter. Further research efforts are needed to increase the body of literature on *C. nodosa*, in terms of its spatio-temporal dynamics, associated drivers, and especially potential links with the ongoing climate change, and its provided ecosystem services.

As regards to the harness of the variety of existing satellite data, numerous studies have assessed seagrass dynamics in a plethora of spatial and temporal scales. In one of the first and most important seagrass change detection assessments, Dekker et al. (2005) exploited four Landsat 5 and 7 images spanning a total of 14 years to map four seagrass species, including ones with great ecologically sensitivity, in Wallis Lake, an estuarine lake in Australia. Pu et al. (2014) also used Landsat 5 data to evaluate seagrass dynamics between 2003 and 2005 in Florida coast. While the two studies monitored change detection of seagrass extent and cover, respectively, Roelfsema et al. (2014) mapped seagrass species, cover and above ground biomass processing a 142-km^2^ time-series of high spatial resolution WorldView-2, IKONOS and Quickbird imagery between 2004 and 2013 with an object-based approach in Moreton Bay, Australia. In another yet exploitation and longest, to the best of our knowledge, of the Landsat archive, Lyons et al. (2012) developed an object-based approach to assess seagrass extent between 1972 and 2010 in Moreton Bay as well. The common denominator of the aforementioned four time-series studies is that they assessed shallow waters up to 7 m in contrast to the 16.5-m deep limit in our study. They also all highlight the significance of remote sensing time series of seagrass habitats for seagrass ecology. Roelfsema et al. (2014) argued that the study of the correlation of seagrass-related physicochemical parameters like water quality and temperature with seagrass distribution and composition is vital. Future approaches to this direction could benefit time-series studies and more broadly the management and conservation of seagrasses.

Generally, there is a need for the development of an automated workflow which would exploit the great quantity of remote sensing information and develop time series of seagrass distribution and other management-related parameters in a time- and cost-efficient as well as accurate fashion. This workflow would enable fast assessment of problematic areas (areas of existing or ongoing regression) and raise the need for appropriate management and conservation measures. As mentioned in the Introduction section, however, to achieve accurate time series of seagrass and broadly coastal submerged habitats, one has to select suitable classifier(s) to the subject in study, as classification of these habitats always precede time series analysis.

### Classification of submerged habitats

We selected Random Forests to solve the classification problem of discriminating between four habitats in the optically shallow waters of the Thermaikos Gulf, NW Aegean Sea. We run the classification experiments on atmospherically and water-column corrected RapidEye reflectance composites using 100 trees which yielded better quantitative results than other numbers of trees. We also used 50 data for each habitat for both the classification and validation to avoid possible overestimation of any of the classes (Traganos and Reinartz, 2017). It is noteworthy, as Figure 1 shows, that we chose training data only from the southeastern part of our survey site and validation data only from the northwestern part of our survey site. This could have led to biased classified results.

Overall, as reported in the error matrices of Table 2, RF exhibited high accuracies in classifying and identifying both seagrass species and especially *P. oceanica* species, up to 98% and 100% producer and user accuracy, respectively. The 5-m pixel-based random forest classification of *P. oceanica* and *C. nodosa* species displayed slightly worse producer accuracies, but higher user accuracies than similar efforts using Sentinel-2A 10-m imagery in the same waters (Traganos and Reinartz, 2017). Particularly for the latter species, its sparse and mixed nature with sandy beds inhibits classification and identification approaches causing the so-called “mixed” pixels. Higher than 5-m resolution approaches employing linear unmixing models and/or object-based classifications could solve this classification issue.

We chose RF to classify and identify Mediterranean seagrasses to achieve more accurate results than simpler, theoretically, algorithms like Maximum Likelihood (Traganos and Reinartz, 2017) which has had a wide application history in the literature of both single- and multi-date studies of seagrass ecosystems (Dekker et al., 2005; Pasqualini et al., 2005; Pu and Bell, 2013; Pu et al., 2014). MLC assumes a normal distribution of classes which is rare in the nature of the examined classes, thus producing inferior results to the more sophisticated machine learning classifiers (Traganos and Reinartz, 2017). RF produced promising results concerning classification of seagrasses recently in two studies (Zhang, 2015; Traganos and Reinartz, 2017). (Zhang et al., 2013) demonstrated the advantage of RF over MLC using hyperspectral imagery. Utilizing 150 trees to run the RF-based experiments, Zhang (2015) achieved better accuracies than the machine learning classifiers of Support Vector Machines (SVMs) and *k-*Nearest Neighbor (*k-*NN) in identifying patchy seagrass in a 40-km^2^ area in lower Florida Keys, but slightly lower accuracy for continuous seagrass. Traganos and Reinartz (2017) compared RF, SVMs and MLC classifiers in a small section of the surveyed site in the present study. They showed that both RF and SVMs performed evidently better than MLC on classifying *P. oceanica* and *C. nodosa* habitats. In the same study, both RF and SVMs displayed lower accuracies on the classification of *C. nodosa* than *P. oceanica* due to the smaller number of field data for the former in addition to its mixed ground with sand.

In summary, machine learning classifiers like RF and SVMs gain more and more interest in coastal habitat remote sensing and, more broadly, in the remote sensing literature (Gislason et al., 2006; Mountrakis et al., 2011). Deep learning techniques concerning submerged habitats are still in their infancy (Call et al., 2003; Calvo et al., 2003) and it is still unknown whether the extra processing power and time to design the experiments are worthy for the potentially better identification that they would offer than machine learning classifiers.

### Pre-classification steps in detection, mapping, and time series of submerged habitats

The steps which precede the classification and subsequent development and analysis of the time series of submerged habitats include geometric, atmospheric, and water column corrections of satellite data in addition to developing satellite-derived bathymetry. In the present study, geometric corrections were already done in the Level 3A RapidEye imagery which we processed in the time series. The FLAASH module, implemented for the atmospheric correction, has been already deployed by several studies for studying the change detection of underwater habitats (Lyons et al., 2010, 2011; Pu and Bell, 2013; Roelfsema et al., 2014).

Regarding the water column correction, the analytical model of Maritorena et al. (1994) accurately retrieved bottom reflectances of both *P. oceanica* and *C. nodosa* seagrass species in the RE time series of the present study. In the same area, Traganos and Reinartz (2017) employed successfully the same model to perform water column corrections for the mapping of the two same species. Dierssen et al. (2003) discussed the good agreement of the model's calculated bottom reflectances with *in situ* ones over dense *Thalassia testudinum* beds in contrast to overestimated seabed reflectances over beds of the same species of sparse to intermediate density up to depths of 9 m in Lee Stocking Island, Bahamas. In addition, Pu et al. (2014) conducted water column corrections following the same analytical model to identify three seagrass species (*T. testudinum, Syringodium filiforme*, and *Halodule wrightii*) in depths up to 4 m in Florida, USA. A similar to ours image pre-processing and processing methodological approach to seagrass change detection led to 14% improved overall accuracies than studies which used analogous data. In another application of Maritorena et al. (1994) water column correction model, Dekker et al. (2005) mapped the change detection of *Posidonia australis, Halophila ovalis, Zostera capricorni*, and *Ruppia megacarpa* in the waters of Wallis Lake in Australia, in depths of <3 m. In antithesis to the field optical measurements of the latter study, we used and developed image-based estimations of both the diffuse attenuation coefficient and infinitely deep water column reflectance. Future *in situ* optical measurements are expected to increase accuracies in water column corrections and succeeding classifications and time series analyses, however, these measurements would also raise the cost of the given study.

Regarding the satellite-derived bathymetry, we created a 5-m resolution bathymetry map of the Thermaikos Gulf using RapidEye imagery to aid the RE-based time series of the two Mediterranean seagrass beds. We should note here that we employed the closest imagery to our *in situ* depth estimations, RE16, to estimate bathymetry for all four images. Traditionally, remote sensing scientists have calculated depth in optically shallow regions by using the band ratio (Lyzenga, 1978). Employing the ratio of blue to green, but in the different central wavelengths of Sentinel-2, 490 and 560 nm, than in the present study, Traganos and Reinartz (2017) developed a bathymetry model for a subsection of the survey site of the present study. They also applied a low pass filter before the estimation of the bathymetry in contrast to the present study where we applied a low pass filter after mapping depth. The lower wavelength of the blue band of RapidEye at 475 nm, however, in comparison to the 490 m of Sentinel 2 is anticipated to produce more accurate bathymetry estimation due to the higher penetration of the blue band in the water column in this case. Moreover, we chose to convert at-water surface reflectances to the remote sensing reflectances for the development of the bathymetry model as the latter are considered more robust to interactions in the air-water interface and water column constituent composition (Dierssen et al., 2003; Dekker et al., 2011).

The dense canopy and the incidental shading produce the often lower than the adjacent optically deep waters reflectance of *P. oceanica* seagrass (Dekker et al., 2006). This issue imposes a problem to accurate bathymetry estimations over this type of seabed. Traganos and Reinartz (2017) overcome this problem by modifying the widely utilized bathymetry algorithm of Stumpf et al. (2003) which displayed negative values over *P. oceanica* beds in relevant experiments. It is worth noting that due to the lack of extensive *in situ* depth data, we tuned our polynomial algorithm using data, mainly from the southeasternmost part of the Thermaikos Gulf. Nevertheless, as we presented in section Pre-classification Steps, we chose *in situ* depth data over all four habitats, namely the two seagrasses, sand and rocky seabed with algae, achieving an accuracy of 91% in the development of the site-specific depth algorithm and a r-squared value of 0.86 with a RMSE of 2.6 m in the validation of this site-specific bathymetry.

Other studies have used either existing pixel-based depth maps produced with acoustic equipment (Pu and Bell, 2013; Pu et al., 2014), have developed their own satellite-derived bathymetry maps implementing either linear or ratio algorithms (Lyons et al., 2011) or have run simulation experiments of the bathymetry effects using HYDROLIGHT, a robust radiative transfer model. It would be interesting to compare in the future bathymetries derived from all of the above sources to study how accuracies deviate in turn.

## Conclusions

The present study demonstrates an off-the-shelf methodology to quantitatively assess the spatio-temporal dynamics of seagrasses and other submerged habitats in clear and homogeneous optically shallow waters using Planet's RapidEye time series of four 5-m satellite images. The methodology includes three stages: (a) pre-processing including atmospheric and water column correction of the satellite data along with satellite-derived bathymetry, (b) machine learning classification using the Random Forest algorithm, and (c) interannual change detection which is presented here as a change of area and associated trend. We applied these three steps to study the dynamics of two Mediterranean seagrasses, *P. oceanica* and *C. nodosa*, in the waters of the Thermaikos Gulf (NW Aegean Sea, Greece) between 2011 and 2016. Total seagrass area has increased by 6.3% at a rate of +6.8 ha/yr, while *P. oceanica* seagrass has regressed by 4.1% at a rate of −11.2 ha/yr and *C. nodosa* seagrass has progressed by 17.7% at a rate of +18 ha/yr throughout the 5 years. In some occasions, *C. nodosa* has been studied to substitute the regressed beds of *P. oceanica*. The aforementioned trends, especially in terms of the regression of the *P. oceanica*, are in line with the reported regression of this valuable seagrass elsewhere in the Mediterranean. This study is the first to report spatio-temporal dynamics of both seagrasses in large scales using remotely sensed data. The remote sensing of seagrasses lying in optically shallow waters (where the observed surface reflectance contains signal from the bottom in contrast to an optically deep column) faces a plethora of inherent obstacles due to the complex nature of the media above the seagrass beds themselves. Obstacles like water column constituents, sunglint, and skyglint presence, air-water interface interference could impede the detection of seagrasses and require, usually, consideration through relevant algorithms. The presented methodological workflow could act like an alternative ecological assessment showing current trends, revealing regressing seagrasses, and allowing better conservation of these complex but also significant ecosystems. Potential improvements in the given approach could be the existence of *in situ* optical measurements of several relevant parameters, broader bathymetry field data, advanced radiative transfer simulations, possible comparison of different machine learning algorithms for the improvement of classification and identification of seagrasses and better tuning of those algorithms. Currently, seagrasses are decreasing in alarming rates in a global scale. Linkage of this decreasing trend with the anthropogenic and natural interference through Earth observation of climate change, eutrophication, coastal development as well as temperature, salinity, and hydrodynamic change could develop and refine machine learning models to ecologically assess seagrass status worldwide. Harnessing the wealth of Earth observation data that this century offers and state-of-the-art machine learning algorithms, we could better understand the thresholds of different seagrass habitats in different aquatic environments and strengthen their conservation management, allowing a brighter future for these significant ecosystem service suppliers.

## Author contributions

DT conceived and designed the study, collected the *in situ* data, performed data analysis and interpretation, and drafted the article; PR supervised the whole study, from the conception to the drafting and critical revision.

### Conflict of interest statement

The authors declare that the research was conducted in the absence of any commercial or financial relationships that could be construed as a potential conflict of interest.

## Footnotes

^1^https://www.planet.com/products/satellite-imagery/files/160625-RapidEye%20Image-Product-Specifications.pdf.

^2^National Statistics Service of Greece, 2011.

^3^National Statistics Service of Greece, 2015.
